# Genome-wide differential gene expression in immortalized DF-1 chicken embryo fibroblast cell line

**DOI:** 10.1186/1471-2164-12-571

**Published:** 2011-11-23

**Authors:** Byung-Whi Kong, Jeong Yoon Lee, Walter G Bottje, Kentu Lassiter, Jonghyuk Lee, Douglas N Foster

**Affiliations:** 1 Department of Poultry Science, Center of Excellence for Poultry Science, University of Arkansas, Fayetteville, Arkansas 72701, USA; 2 Department of Chemistry, Purdue University, West Lafayette, IN 47907, USA; 3 Department of Animal Science, University of Minnesota, St. Paul, MN 55108, USA

## Abstract

**Background:**

When compared to primary chicken embryo fibroblast (CEF) cells, the immortal DF-1 CEF line exhibits enhanced growth rates and susceptibility to oxidative stress. Although genes responsible for cell cycle regulation and antioxidant functions have been identified, the genome-wide transcription profile of immortal DF-1 CEF cells has not been previously reported. Global gene expression in primary CEF and DF-1 cells was performed using a 4X44K chicken oligo microarray.

**Results:**

A total of 3876 differentially expressed genes were identified with a 2 fold level cutoff that included 1706 up-regulated and 2170 down-regulated genes in DF-1 cells. Network and functional analyses using Ingenuity Pathways Analysis (IPA, Ingenuity^® ^Systems, http://www.ingenuity.com) revealed that 902 of 3876 differentially expressed genes were classified into a number of functional groups including cellular growth and proliferation, cell cycle, cellular movement, cancer, genetic disorders, and cell death. Also, the top 5 gene networks with intermolecular connections were identified. Bioinformatic analyses suggested that DF-1 cells were characterized by enhanced molecular mechanisms for cell cycle progression and proliferation, suppressing cell death pathways, altered cellular morphogenesis, and accelerated capacity for molecule transport. Key molecules for these functions include E2F1, BRCA1, SRC, CASP3, and the peroxidases.

**Conclusions:**

The global gene expression profiles provide insight into the cellular mechanisms that regulate the unique characteristics observed in immortal DF-1 CEF cells.

## Background

Normal (primary) cultured cells derived from living tissue exhibit a limited life span reaching replicative senescence in a non-dividing state [1]. Each cell division results in the generation and accumulation of various cellular genetic alterations, such as telomere shortening caused by the inability of DNA polymerases to fully replicate the ends of linear chromosomes [2,3]. This inability to overcome these alterations ultimately leads to cellular aging. Most cells are unable to overcome senescence unless key tumor suppressor pathways are first altered. Thus, cellular immortalization has been achieved by genetic alterations which bypass the stages leading to cellular senescence.

Spontaneous immortalization is a rare event in human and avian cells, but occurs much more frequently in rodent cells [4]. Unlike virally or chemically induced tumor cell lines, spontaneously induced, non-transformed cell lines lacking endogenous and exogenous viral genomes are much more useful for studying the conversion to an immortal state and to evaluate the effects of viral infection. Traditionally, in the absence of a suitable avian cell line, primary chicken embryo fibroblasts (CEF) have been used in virology and vaccine production, although a major disadvantage is the fluctuation of virus titers from lot to lot. Thus, there are advantages to using a spontaneously immortalized non-transformed cell line for vaccine production, which provides an unlimited supply of identical cells.

**Figure 1:**
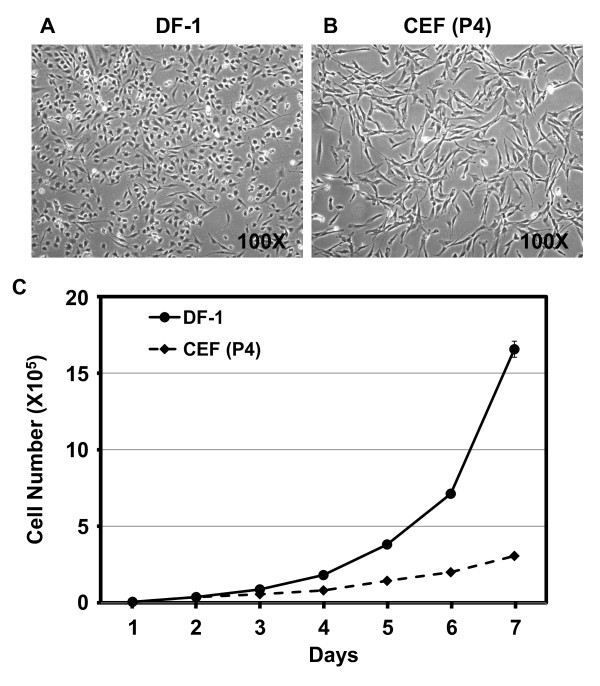
**Morphology and cell growth kinetics for DF-1 and primary CEF (passage 4) cells**. Cell images for DF-1 (P285) (A) and primary CEF (P4) (B) cells were obtained by inverted microscopy at 100× magnification. (C) Growth kinetics. Immortal DF-1 and primary CEF cells were seeded at 1 × 10^5 ^cells per 10-cm dish and the accumulated cell numbers were counted at each day for 7 days. Experiments were repeated three times.

The immortal DF-1 CEF cell line was established spontaneously from Line 0 (endogenous-virus negative; [5]) embryos and has been widely used for the propagation of various avian viruses, including avian sarcoma leukosis virus [6,7], avian leukosis virus [8], Marek's disease virus [9], avian influenza virus [10,11], infectious bursal disease virus [12], and avian metapneumovirus [13,14]. The non-transformed DF-1 CEF cell line has been continuously grown in culture for more than 300 passages and does not harbor any known endogenous viruses [7,15]. DF-1 cells have enhanced growth potential compared to their primary CEF counterparts [6,16]. The morphology of DF-1 cells is that of a typical spindle-shaped fibroblast, but is much smaller than its primary CEF counterpart (Figure 1; [17]).

Various genetic, biochemical, and physiological characteristics of the DF-1 cell line have been reported. At the chromosomal level, DF-1 cells display different ploidy lineages and chromosomal rearrangements by maintaining a complex derivative karyotype which may be caused by chromosome fusions in homozygous and heterozygous conditions. In addition the DF-1 cells contain a greater amount of telomeric sequence repeats per genome compared to normal chicken cells and to a telomerase positive transformed lymphoma cell line [18]. Chromosome rearrangements and different ploidy thus may influence structural or dosage-related alterations in gene expression. Indeed, DF-1 cells retain various genetic alterations of down-regulated p53 function, an up-regulated pRB (retinoblastoma protein) and E2F1 pathway, and elongated telomere length, which are commonly found in immortalized cells [19]. Compared to the parent line of primary CEF cells, DF-1 cells were shown to transcriptionally increase mitochondrial encoding gene expression and elevated mitochondrial respiratory functions, which supports its rapidly dividing cellular characteristics [16]. Moreover, cellular antioxidant genes, such as manganese containing superoxide dismutase (MnSOD or SOD2), copper-zinc containing SOD (CuZnSOD or SOD1), and catalase were deregulated transcriptionally and functionally in the DF-1 cell line. These deregulated antioxidant functions may be considered to be responsible for hypersensitivity to oxidative stress shown in DF-1 cells [20-22].

The DF-1 CEF cell line is a biologically important spontaneously immortalized cell line that has been utilized by great number of research groups for a host of research topics. Due to the diverse utility and the potentially unique genetic characteristics, the DF-1 cell line (along with several other chicken cell lines and chicken breeds) will be subjected to whole genome sequencing within the year (personal communication Jerry Dodgson, Michigan State University).

Biologic, virologic and important genetic alterations in immortal DF-1 CEF cells have been reported, however there are no reports concerning genome-wide gene expression profiling of the DF-1 cells to our knowledge. Thus, the major goal of this study was to conduct global gene expression analysis to profile differentially expressed genes in DF-1 CEF cells compared to their primary CEF counterpart using a 44K chicken oligo microarray. The results indicate that DF-1 cells retain cellular characteristics of enhanced cell cycle progression and proliferation, down-regulated cell death pathways, hyperactive mitochondrial functions, and altered cellular morphogenesis.

## Results and Discussion

### Morphology and growth characteristics of DF-1 CEF cells

Immortal DF-1 CEF cells have been growing continuously in culture for a number of years. DF-1 cells at passage 285 morphologically showed typical characteristics of fibroblast cells, but are clearly smaller in size especially regarding cellular projections compared to primary passage 4 CEF cells (Figure 1A and 1B). Growth rates of DF-1 cells showed between 1.0 to 1.2 population doublings per day (PD/d) compared to 0.6 - 0.8 PD/d of primary CEF cell counterpart (Figure 1C).

### Gene expression profile of immortal DF-1 CEF cells

To find transcriptional alterations in DF-1 cells, genome-wide expression profiling was conducted using RNA from primary and immortal DF-1 CEF samples. Although the primary CEF counterpart cells used in this study (SPF embryo origin) were not the original embryonic cells used for the establishment of DF-1 cells [from Line 0 (endogenous-virus negative) embryo origin], comparison of transcriptional alterations by microarray analysis was conducted to understand cellular characteristics of immortal- and rapidly growing DF-1 CEF cells compared to early passage of primary CEF cells having limited life-span and a relatively slow proliferation rate. Of the 44K probes used in the microarray analysis, a total of 3876 differentially expressed genes were identified in DF-1 CEF cells with a 2 fold level cutoff that included 1706 up-regulated and 2170 down-regulated genes (Additional file 1). To validate the microarray results, 21 randomly chosen genes from the 3876 differential expression list were subjected to qPCR along with the GAPDH loading control gene. Results indicated that increased or decreased expression levels for all genes tested were well-matched in assays between microarray and qPCR analysis (Table 1). When 3876 differentially expressed probes were analyzed using Ingenuity Pathways Analysis (IPA, Ingenuity^® ^Systems, http://www.ingenuity.com), 902 were classified as functionally known genes (Additional file 2). A list of the 10 most up- and down-regulated differentially expressed genes in the DF-1 cells are provided in Table 2.

**Table 1:** Comparison of fold changes between microarray and qPCR

**Accession No**.	Gene Symbol	Microarray	qPCR
AB109635	HMGCR	-1.03	-0.81
AB196971	APCDD1	4.75	7.96
AF505881	SCX	-3.27	-1.94
AJ131110	TWIST2	4.61	6.77
BU279212	CBLN2	7.84	8.64
BX929635	NDP	-4.26	-1.64
BX931599	VIPR2	-2.90	-11.34
BX932694	MPZL2	5.51	4.16
BX933478	MXRA5	-2.69	-7.97
BX933888	C1QTNF3	-2.53	-2.28
BX935456	EGLN3	-3.04	-4.48
BX936211	TMEM116	4.26	5.94
CK611983	CSTA	4.49	7.92
CR385566	CLEC3B	-4.46	-3.58
D87992	ANPEP	-4.25	-6.29
M60853	THBS2	-5.15	-5.28
M64990	PTGS2	-6.78	-10.36
M80584	LUM	-3.14	-4.59
M87294	NPY	-3.73	-7.15
X87609	FST	-4.12	-10.04

The gene expression levels of 20 genes from microarray analysis were confirmed by qPCR. The expression levels were presented by fold changes values in microarray analysis, while, for qPCR, the values were calculated by 2^-ΔΔCT ^method, which were comparable to fold changes in the microarray. All values are mean values determined by the calculations from three replicate assays.

**Table 2:** The 10 most up- and down-regulated genes in DF-1 cells as determined by Ingenuity Pathway Analysis software

The 10 most up-regulated genes				
**ID**	**Symbol**	**Entrez Gene Name**	**Log Ratio**	**p-value**

AB196971	APCDD1	adenomatosis polyposis coli down-regulated 1	4.75	4.68 × 10^-8^
AJ131110	TWIST2	twist homolog 2 (Drosophila)	4.61	3.93 × 10^-9^
BX936211	TMEM116	transmembrane protein 116	4.26	9.58 × 10^-10^
S64689	MYH6	myosin, heavy chain 6, cardiac muscle, alpha	3.52	4.92 × 10^-6^
AJ851540	CARD11	caspase recruitment domain family, member 11	3.51	1.66 × 10^-8^
BX932923	PLEK2	pleckstrin 2	3.47	2.63 × 10^-8^
CR523499	OSGIN1	oxidative stress induced growth inhibitor 1	3.43	5.04 × 10^-9^
DQ129668	COLEC10	collectin sub-family member 10 (C-type lectin)	3.19	1.81 × 10^-7^
BX933215	SOCS1	suppressor of cytokine signaling 1	3.05	3.63 × 10^-7^
CR407099	VPS13B	vacuolar protein sorting 13 homolog B (yeast)	3.03	3.44 × 10^-6^

**The 10 most down-regulated genes**				

ID	Symbol	Entrez Gene Name	Log Ratio	p-value

X59284	NOV	nephroblastoma overexpressed gene	-8.40	3.11 × 10^-9^
AJ719388	SLC25A4	solute carrier family 25 (mitochondrial carrier; adenine nucleotide translocator), member 4	-7.69	1.22 × 10^-10^
Y138247	CDKN2B	cyclin-dependent kinase inhibitor 2B (p15, inhibits CDK4)	-7.68	2.75 × 10^-10^
X80503	ST3GAL1	ST3 beta-galactoside alpha-2,3-sialyltransferase 1	-7.26	4.67 × 10^-9^
M64990	PTGS2	prostaglandin-endoperoxide synthase 2 (prostaglandin G/H synthase and cyclooxygenase)	-6.78	1.11 × 10^-10^
X935556	CTHRC1	collagen triple helix repeat containing 1	-6.78	3.20 × 10^-8^
M68514	EPHA3	ephrin receptor A3	-6.73	5.89 × 10^-9^
AJ719946	MANSC1	MANSC domain containing 1	-6.65	5.66 × 10^-8^
AJ251273	CCK	cholecystokinin	-6.63	1.27 × 10-11
Y049705	MAB21L1	mab-21-like 1 (C. elegans)	-6.44	2.26 × 10^-10^

Fold change (FC) values were indicated by log2. The highly differentially expressed genes were sorted. All genes were matched and verified with UniGene function of NCBI database.

### The 10 up- and down-regulated genes in DF-1 cells exhibiting the greatest differential expression

The 10 most up-regulated genes (Table 3) are related to functions of cell cycle and proliferation, intracellular trafficking, cytoskeletal arrangement, and host-defense mechanisms against pathogenic infections. In contrast, the 10 most down-regulated genes in DF-1 cells (Table 3) are associated with cell cycle arrest and apoptosis, homeostasis, cell shape and movement, cellular respiration, and organ development. Of these, the up-regulation of OSGIN1, COLEC10, and SOCS1 and the down-regulation of ST3GAL1, PTGS2, and CCK in DF-1 cells appears initially to be incongruous with the rapid growth potential that is characteristic of these cells. Although the connections to cellular phenotypes from mRNA expression data may not be as strong as expected due to the potential lack of correlation between mRNA expression and protein abundance (which have been reported previously in both prokaryotes and eukaryotes [23-27]), the highly and dramatically differentially expressed genes may represent correlations between transcripts, protein abundance and the differential abundance of proteins in two different cell types, respectively. Thus, further study is needed to reveal the functional roles of these genes in DF-1 cells.

**Table 3:** Biological functions of the 10 most up- and down-regulated genes

Up-regulation	
**Symbol**	**Functions**

APCDD1	• Directly regulated by the β-catenin/T-cell factor signaling complex • Increased expression in colon cancer cells and potential roles in colorectal tumorigenesis [75].

TWIST2	• A basic helix-loop-helix transcription factor • Inhibits both p21WAF1/Cip1 (cyclin dependent kinase inhibitor) and muscle creatinine kinase (MCK) • Functional association with cellular growth arrest and myogenesis specific expression [76] • Possibly alternate regulatory factor involved in the lowered expression of p21WAF1/Cip1 in DF-1 cells that in turn would support the faster growth rate that is characteristic of the DF-1 cell line [19].

TMEM116	• Unknown functions • A single nucleotide polymorphisms are associated with insulin-dependent diabetes mellitus in human [77]

MYH6	• Alpha subunit of cardiac muscle myosin • Association cytoskeletal or muscle architecture [78] • Taken together with MYL10 (myosin light chain 10), which is up-regulated in DF-1 cells (Additional file 2), mutations are associated with cardiomyopathy or cardiac hypertrophy [79]

CARD11	• Known as Carma1 belonging to the membrane-associated guanylate kinase (MAGUK) family that is essential in antigen receptor-induced nuclear factor κB (NF- κB) activity in T-cell activation [80]

PLEK2	• A membrane associated protein containing PH (pleckstrin homology) motifs that bind polyphosphoinositides • Roles in orchestrating cytoskeletal structural arrangement [81]

OSGIN1	• Known as BDGI [BMSC (bone marrow stromal cell)- derived growth inhibitor] or OKL38 (pregnancy-induced growth inhibitor) • Suppression of the growth of MCF7 human breast cancer cell by inducing cell cycle arrest and apoptosis when OSGIN1 was ectopically expressed exogenously [82]

COLEC10	• Known as CLL1 (colletin liver 1), a member of the C-lectin family • Roles in initial host defense by binding sugars on the cell surface of microorganisms through their carbohydrate recognition domain [83]. • Essential host factor for early replication of influenza virus in cultured cells revealed by genome wide siRNA screening [84] • Its mRNA down-regulated in human liver hepatocellular carcinoma by microarray analysis [85].

SOCS1	• Suppression of many cytokine-signaling pathways by inhibiting JAK tyrosine kinase activity and functions as antioncogene by antagonizing tumor cell growth [86].

VPS13B	• Known as COH1, involved in intracellular vesicle-mediated sorting and transport of proteins and with Cohen syndrome, which is an autosomal recessive disorder in human caused by the genetic mutation in COH1 gene [87].

**Down-regulation**	

Symbol	Functions

NOV	• Known as CCN3, a member of the secreting insulin like growth factor binding protein family with antiprolifereative effects on tumor cells [88,89].

SLC25A4	• Known as ANT1 (adenine nucleotide translocator 1) • Localized to the inner mitochondrial membrane, exchanges cytosolic ADP for mitochondrial ATP, and induces apoptosis by the mitochondrial recruitment of NF-κB [90]. • Decrease in ANT1 might play in a role in immortalization characteristics by suppressing the induction of apoptosis in DF-1 cells.

CDKN2B	• Known as p15INK4B, CDK4/6 inhibitor • Arrest cell cycle and induce cellular senescence [91]. • The down-regulation in DF-1 cells caused by hyper-methylation • Functional roles both in the progression of cellular senescence and in brain development were reported previously [92,93].

ST3GAL1	• Type II membrane protein that catalyzes the transfer of sialic acid from CMP-sialic acid to galactose-containing substrates • A knock-out mutation increases apoptosis of mouse T lymphocytes expressing the CD8 complex, indicating a homeostatic function of STGAL1 in T lymphocyte [94].

PTGS2	• Known as COX2 (cyclooxygenase 2), the key rate-limiting enzyme in prostaglandin biosynthesis with both dioxygenase and peroxidase activity • Inhibition of apoptosis by suppressing caspase pathways and the increase of survival mechanisms through Akt activation [95].

CTHRC1	• A secreted protein in injured and diseases arteries that inhibits collagen expression and promotes cell migration [96].

EPHA3	• A unique member of the receptor tyrosine kinase family • Roles in regulating cell shape and cell movement [97].

MANSC1	• A protein containing a MANSC (motif at N terminus with seven cysteines) domain that presents certain membrane- and extracellular proteins such as LPR11 (low-density lipoprotein receptor-related protein 11) and HAI1 (hepatocyte growth factor activator inhibitor 1) [98] • Unknown the functions

CCK	• A gastrointestinal and neuronal peptide with important regulatory roles in the digestive tract and nervous system, including both acute and more chronic trophic effects • Binding its receptor triggers the activation of multiple signal transduction pathways that relay the mitogenic signal to the nucleus and promote cell proliferation [99].

MAB21L1	• A knock-out mutation led to the defects in eye and perputial gland formation [100]

### Functional groups of differentially expressed genes

**Figure 2:**
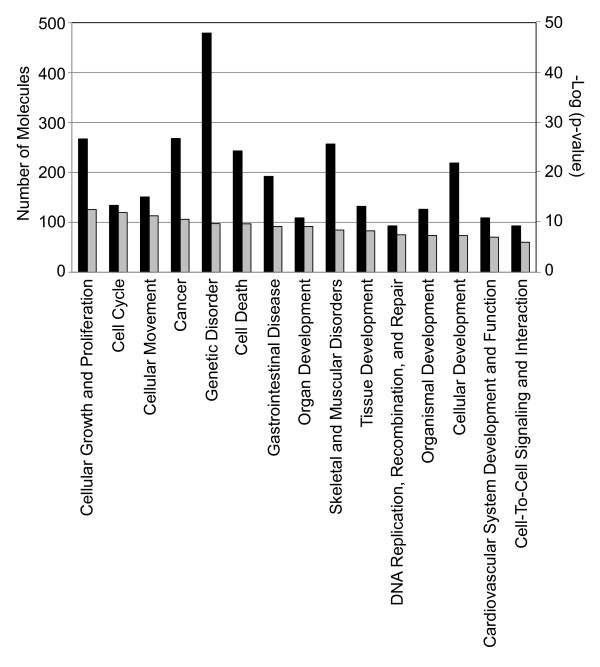
**Functional Gene Ontology (GO) for differentially expressed genes**. The 904 genes were categorized into functional groups by the IPA program. Closed bars represent the number of probes for each cluster, while gray bars indicate -log(p-value), which was calculated by IPA software showing the levels of relatedness. The left Y-axis shows the total number of probes for each biological functions, the right Y-axis shows the -log(p-value), and the X-axis indicates name of functions.

The IPA program generated bioinformatics data sets including functional groups (gene ontology; GO) and gene networks for differentially expressed genes in immortal DF-1 CEF cells. Of the biologically functional groups for 904 differentially expressed genes, the top 15 functional groups are displayed in Figure 2. The greatest numbers of genes are mainly categorized into functionalities of cellular growth and proliferation, cell cycle, cellular movement, cancer, genetic disorders, and cell death, suggesting that the transcriptional alterations that occurred in DF-1 cells are closely related and likely responsible for a great deal of the rapid growth and phenotypic changes in this cell line.

### Gene networks

**Figure 3:**
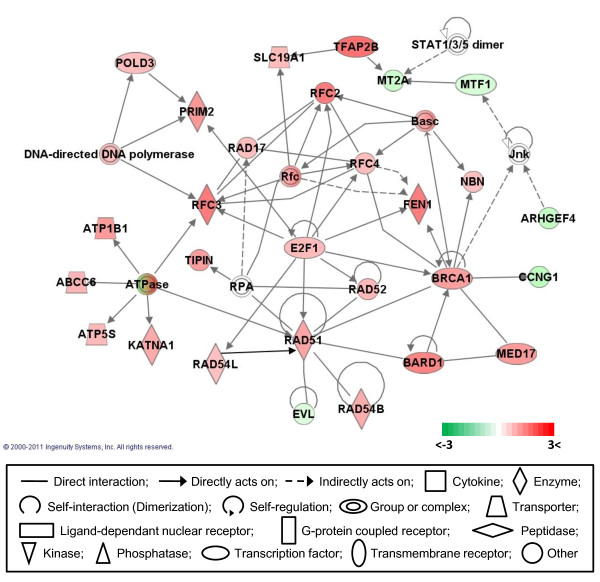
**Gene network #1**. Molecular interactions among important focus molecules are displayed. Green represents down-regulated genes while red depicts up-regulated genes. White symbols indicate neighboring genes, that are functionally associated, but not included in the differentially expressed gene list. The intensity of color represents the average of log fold change in a given population. The numbers below the color change bar denote log_2 _values. Symbols for each molecule are presented according to molecular functions and type of interactions.

**Figure 4:**
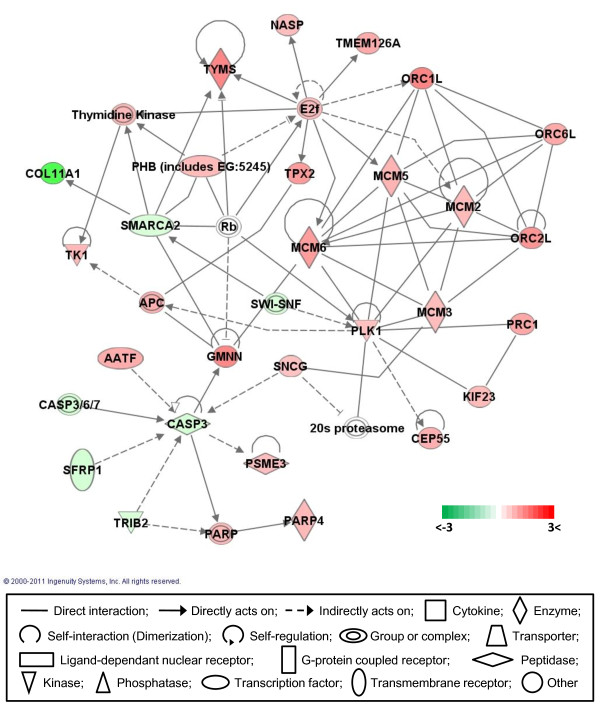
**Gene network #2**. Molecular interaction, symbols, and color schemes are the same as the description in Figure 3.

**Figure 5:**
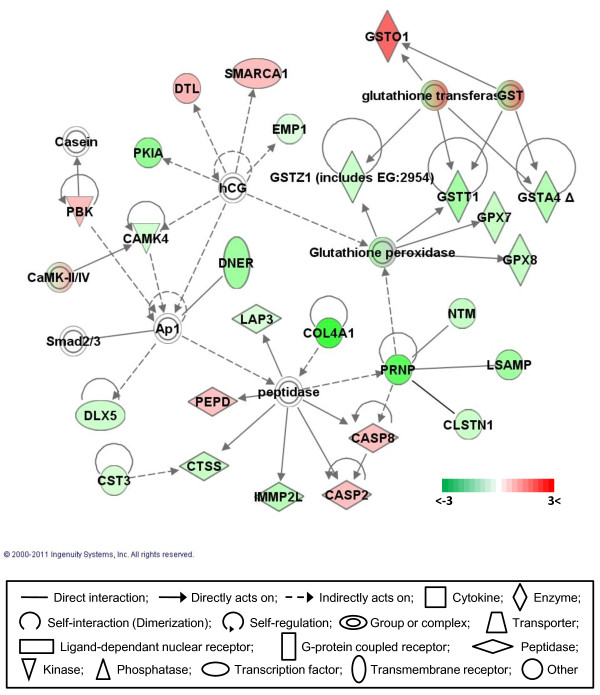
**Gene network #3**. Molecular interaction, symbols, and color schemes are the same as the description in Figure 3.

**Figure 6:**
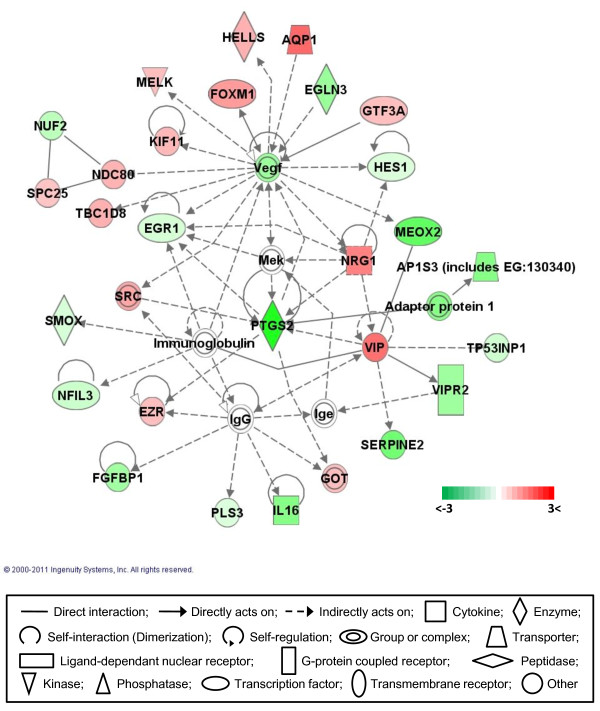
**Gene network #4**. Molecular interaction, symbols, and color schemes are the same as the description in Figure 3.

**Figure 7:**
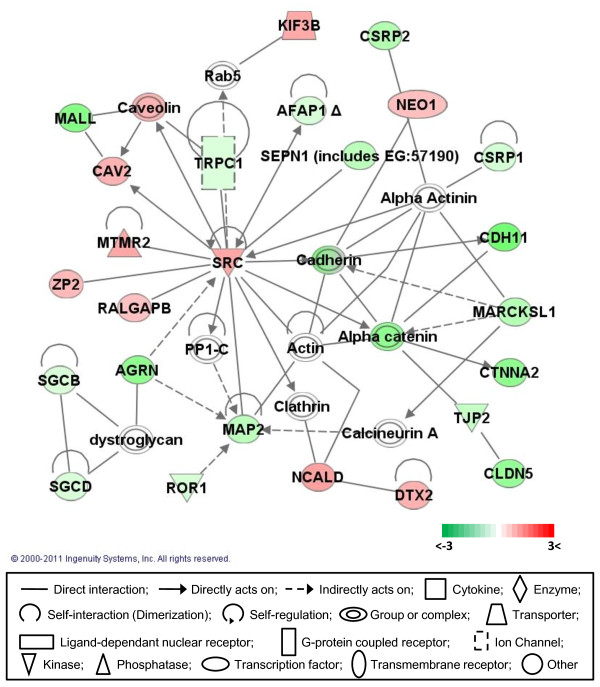
**Gene network #5**. Molecular interaction, symbols, and color schemes are the same as the description in Figure 3.

Gene network analysis, which represents the intermolecular connections among interacting genes based on functional knowledge inputs, was performed on the differentially expressed genes using the IPA program. Of various assay settings, the simplest settings (35 focus molecules and 10 networks) were employed to analyze molecular gene networks in order to facilitate and summarize the connections among the larger number of differentially expressed genes (Table 4 and Figures 3, 4, 5, 6, 7). A discussion of the top five gene networks is provided below and gene information for focus molecules in each network was listed in Additional file 3.

**Table 4:** Associated network functions

ID	Associated network functions	Score	Focus Molecules
1	Cell Cycle, DNA Replication, Recombination, and Repair, Cellular Assembly and Organization	33	28

2	DNA Replication, Recombination, and Repair, Cell Cycle, Cellular Assembly and Organization	31	27

3	Cancer, Cardiovascular System Development and Function, Organismal Development	30	26

4	Molecular Transport, Tissue Morphology, Cell Cycle	29	27

5	Cellular Assembly and Organization, Developmental Disorder, Skeletal and Muscular Disorders	28	25

Functions associated with 10 networks are listed. Score means the number of network eligible molecules out of differentially expressed genes.

Network #1 is closely associated with the E2F1 and BRCA1 (breast cancer 1, early onset) pathways in cell cycle regulation (Figure 3). Likewise, the top functions related to network #1 are cell cycle regulation, DNA replication, recombination, repair, and cellular assembly and organization. Up-regulation of E2F1 in immortal CEF cell lines including DF-1, heart derived- and breast derived CEF cells was reported previously together with the genetic alterations for cell cycle regulatory genes, such as the down-regulation of p53, MDM2, p21CIP as well as the upregulation of pRB, the cyclins (except cyclin D2), c-Fos, c-Jun, and Bcl2 [19]. These cell cycle regulatory genes showed a similar expression pattern in the full differential expression list of 3876 genes. However, with the exception of E2F1, these genes were not recognized by the IPA program. This difference might be the result of the IPA program, which focuses mainly on mammalian gene information and pathways, and does not fully cover chicken gene annotations. It suggests that comparing and contrasting data of both the IPA differential expression list and the manufacturer's differential expression list is more helpful for comprehensively understanding the differential expression datasets.

E2F1 is a member of the E2F family of transcription factors that play a crucial role in controlling the cell cycle by association with the tumor suppressor protein, pRB (retinoblastoma protein). E2F transcription factors are the effectors of the G1/S transition of the cell cycle. When bound to DNA, E2F transcription factors exist either as free E2F/DP (E2F dimerization partner) heterodimers, or are associated in larger complexes containing members of the retinoblastoma family (pRB, p107, p130) and members of the cyclin/CDK protein families. CDKs are comprised of a family of serine/threonine protein kinases that phosphorylate a number of substrates mainly implicated in cell cycle progression and transcription. Association of E2Fs with the pRB family facilitates active repression through recruitment of histone deacetylases [28]. Genes directly interacting with E2F1 include factors for DNA replication [e.g. DNA replication factor C (RFC) 2, 3, 4] [29], DNA recombination and repair of double strand breaks (e.g. RAD 17, 51, 52, 54B, 54L and FEN1; flap structure-specific endonuclease 1) [30,31], and maintenance of genomic stability (e.g. BRCA1). BRCA1 is a nuclear phosphoprotein that makes a super complex, denoted as Basc (BRCA1 associated genome surveillance complex), which is associated with tumor suppressors and DNA damage repair proteins such as nibrin (NBN) [32]. In network #1, BRCA1 binding proteins, MED17 (mediator complex subunit 17), which is a transcription co-factor with TFIID, RAD51 and BARD1 (BRCA1 associated RING domain 1) are also up-regulated, suggesting that BRCA1 transcriptional activity may be increased in DF-1 CEF cells. Also DNA polymerases including POLD3 (polymerase-DNA directed-delta 3 subunit) and PRIM2 (primase 2) were up-regulated in network #1. The up-regulation of all of these genes and complexes make 'teleological sense' with regard to the rapid growth characteristics of DF-1 CEF cells. In addition, ATPase related factors including ATPB1 (ATPase, Na+/K+ transporting), ABCC6 (ATP binding cassette, sub-family 6), ATP5S (ATP synthase, H+ transporting, mitochondrial complex), and KATNA1 (ketanin p60- ATPase containing subunit A1) were also up-regulated and consistent with the previous report of the hyperactivation of mitochondrial functions and up-regulation of mitochondrial gene expression [16]. These results suggest that DF-1 CEF cells may contain cellular systemic alterations to accelerate metabolic energy expenditure. Moreover, cellular proliferation inhibitory factors or apoptosis inducing factors, such as MT2A (metallothionein 2A) and ARHCEF4 (Rho guanine nucleotide exchange factor 4) were down-regulated in DF-1 CEF cells [33,34] that again would contribute to the phenotypic expression of rapid growth rate and resistance to senescence that is characteristic of DF-1 cells.

Similar to network #1, the top functions of network #2 include DNA replication, recombination and repair, cell cycle, and cellular assembly and organization (Figure 4). Central in this network are CASP3 (caspase 3), molecules inducing apoptosis including SFRP1 (secreted frizzled-related protein 1) and TRIB2 (tribbles homolog 2) which were all down-regulated. The Caspase family are cysteine-aspartic acid proteases which play major roles in the execution phase of cell apoptosis [35,36]. SFRP1 acts as soluble modulators of Wnt (hybrid of wingless and integration 1) signaling and suppresses tumor cell growth through the Wnt signaling pathway [37]. 'However, functional roles of SFRP1 in apoptosis related to CASP3 expression depends on the cell type [38]. TRIB2, which is an atypical protein serine-threonine kinase, is known to coordinate cell proliferation, migration, and morphogenesis during the development of Drosophila and Xenopus embryos and is involved in apoptosis in mammalian cell lines [39]. Also, AATF (apoptosis antagonizing transcription factor), which suppresses apoptosis induced by oxidation [40], was up-regulated in DF-1 CEF cells. These results generally suggest cell death pathways are suppressed in DF-1 cells that, in turn, support the immortal and hyperproliferative capability of DF-1 cells. Moreover, in human, GMNN (geminin), which negatively regulates DNA replication 'licensing' (the one time initiation of replication in a single cell cycle) by preventing the formation of the pre-replicative complex on origins of replication through the physical association with the 'licensing' factor Cdt1 [41,42], was cleaved by CASP3 during apoptosis [43]. The suppression of GMNN by siRNA knockdown can selectively kill cancer cells [44] and GMNN directly interacts (binds to) MCM6 (minichromosome maintenance complex component 6), which is an essential factor for the initiation of eukaryotic genome replication [45] in addition to other MCM molecules including MCM2, 3, 5 [46,47] in network #2, indicating that GMNN may have an important role in expediting DNA replication. Other E2F-1 transcription targets including TYMS (thymidylate synthetase), NASP (nuclear autoantigenic sperm protein-histone binding), ORC1L (origin recognition complex-subunit 1-like), and TPX2 (microtubule-associated, homolog) are up-regulated in DF-1 cells [48,49]. Furthermore, direct interactions among highly expressed ORC1L, ORC6L, ORC2L and MCM families, which are components protein complex essential for the initiation of the DNA replication in eukaryotic cells, were found in network #2 [50,51]. Up-regulation of PLK1 (polo like kinase1), KIF23 (kinesin family member 23), and PRC1 (protein regulator of cytokinesis 1) through the direct interactions among those molecules may facilitate mitosis and cytokinesis at the late phase of the cell cycle in DF-1 cells [52-54].

Molecules in network #3 are involved in cancer, cardiovascular system development and function, and organismal development (Figure 5). Glutathione peroxidases (GPX7 and 8) and glutathione S transferases (GST-A4, -T1, and -Z1) were downregulated except for GST-O1. This result is consistent with the previous report of higher levels of reactive oxygen species that were produced and accumulated in DF-1 CEF cells possibly due to higher respiratory rates, higher superoxide dismutase and lowered catalase activity, resulting in hypersensitivity to oxidative damage [22]. Taken together, lower levels of GPXs and GSTs may be responsible for higher levels of intracellular oxidative stress and susceptibility to oxidative damage in DF-1 cells. In contrast to the down-regulation of CASP3 in network #2, initiator caspases (CASP) 2 and 8 were up-regulated in DF-1 cells implicating a more active induction of apoptosis when DF-1 cells encounter cell damage, such as oxidative stress or pathogenic infections. DF-1 cells have been utilized as an excellent substrate for the propagation of various viruses, since the cells support high virus titers and generate clear cytopathic effects, such as syncytium formation (nuclear aggregation during virus replication) [6,7,9,10]. Virus infections usually induce apoptosis during lytic propagation stages. Therefore, the up-regulation of apoptosis initiator caspases may play a role during virus infection in DF-1 cells. In addition, prion protein (PRRN) and its interacting proteins including NTM (neurotrimin), LSAMP (limbic system-associated membrane protein), and CLSTN1 (calsyntenin 1) were down-regulated in DF-1 cells. Normally, functional cellular PRRN is known to promote G1/S cell cycle processing, and has resulted in increasing proliferation of human gastric cancer cells [55], but the higher proliferation capability of DF-1 cells may not be stimulated by prions and their interacting molecules.

Network #4 contains molecules involved in molecular transport, tissue morphology, and the cell cycle (Figure 6). In network #4, three subunits of the NDC (nuclear division cycle) 80 complex, which is a homolog of Yeast kinetochore complex component containing NDC80, (a. k. a. HEC1 in mammalian species), SPC (spindle pole component) 24, SPC25, and NUF2, and mediates attachment of chromosomes to microtubules, were differentially expressed showing up-regulation of NDC80 and SPC25 and down-regulation of NUF2. The NDC80 complex is evolutionarily conserved and contains four subunits SPC24, SPC25, NUF2 and NDC80. In budding yeast, the NDC80 complex plays a critical role in establishing the stable kinetochore-microtubule interactions required for chromosome segregation in mitosis [56,57]. Of the NDC80 subunits, chicken SPC24 has not been characterized to date. The unbalanced expression of the components in the NDC80 complex in DF-1 cells may lead unstable chromosome segregation, resulting in different ploidy and a complex derivative karyotype caused by chromosomal rearrangements during rapid proliferation cycles [18]. Several secreting proteins, including IL16 (interleukin 16), FGFBP1 (fibroblast growth factor binding protein 1), SERPINE2 (serpine peptidase inhibitor), VIP (vasoactive intestinal peptide), and NRG1 (neuregulin 1) are found in network #4. VIP and NRG1 were up-regulated, while FGFBP1, IL16 and SERPINE2 were down-regulated. VIP is known to increase cyclin D1 expression and cell proliferation [58], and NRG1, which is a ligand for epidermal growth factor and its receptor, causes constitutive activation of several signaling pathways, such as the Erk1/2, Erk5, and Akt routes, which have been linked to cell proliferation [59]. SERPINE2, a.k.a thrombin inhibitor protease nexin 1, has negative effects on myofibroblastic cell growth by regulating PI3 kinase-Akt pathway [60]. The ectopic expression of the IL16 prodomain in tumor cell lines has triggered growth arrest and apoptosisis [61]. With the exception of FGFBP3, differentially expressed secreted proteins in DF-1 CEF cells were closely related to growth promoting activity. Factors involving tissue morphology in network #4 include EGLN3 (egl nine homolg 3), EGR1 (early growth response 1), FOXM1 (forkhead box M1), HES1 (hairy and enhancer of split 1, Drosophila), MEOX2 (mesenchyme homeobox 2), NFIL3 (nuclear factor, interleukin 3), NRG1, PTGS2 (prostaglandin-endoperoxide synthase 2), VIP, and VIPR2 (VIP receptor 2). The specific functional roles of differentially expressed genes in the morphogenesis of DF-1 cells are being further investigated.

Finally, molecules in network #5 are involved in the cellular assembly and organization, developmental disorders, and skeletal and muscular disorders (Figure 7). Network #5 was mainly centered around up-regulated SRC (v-src homolog - avian) and down-regulated actin related factors. SRC, the cellular homolog of the Rous sarcoma virus v-src, is a protooncogene and may play a role in cell growth in addition to embryonic development. SRC is a tyrosine kinase whose enzymatic activity is necessary to induce oncogenic transformation [62]. The SRC interactive molecules including CAV2 (caveolin 2; [63]), MTMR2 (myotubularin related protein 2; [64]), ZP2 (zona pellucida glycoprotein 2; [65]), RALGAPB (Ral GTPase activating protein-beta subunit; [65]), AFAP1 (actin filament associated protein 1;[66]), and TRPC1 (transient receptor potential cation channel-subfamily C; [67]) are phosphorylated by SRC and are considered to improve cellular proliferation, suggesting that differentially expressed SRC and SRC-interacting molecules may play important roles for the rapid proliferation of DF-1 cells. Cellular structural proteins such as actinin, cadherin, and catenin, and their interacting molecules including CSRP (cystein and glycine-rich protein) 1, CSRP2, CDH11 (cadherin 11), MARCKSL1 (MARCKS like 1; muscle LIM protein, MLP), CTNNA2 (catenin alpha 2), and TJP2 (tight junction protein 2) were down-regulated in DF-1 cells, suggesting that these factors may be involved in the distinct morphology of DF-1 cells compared to primary CEF cells. Factors involved in cellular assembly and organization in network #5 include AGRN (agrin), MAP2 (microtubule-associated protein 2), SGCB (sarcoglycan beta), SGCD (sarcoglycan delta), and SRC. Proteins functioning in skeletal and muscular disorders in network #5 include CDH11, CSRP2, CTNNA2, MAP2, ROR1 (receptor tyrosine kinase like orphan receptor 1), SEPN1 (selenoprotein 1), SGCB, SGCD, and SRC and factors related to developmental disorder are MARCKSL1, SGCB, SGCD, SRC, and TRPC1. Specific roles of each of these factors need further investigation.

In summary, global gene expression analysis in this study provides insight into the entire genome-wide alterations in immortal DF-1 CEF cells. Bioinformatic analyses suggested that DF-1 cells are characterized by enhanced molecular mechanisms for cell cycle progression and proliferation, suppressing cell death pathways, altered cellular morphogenesis, and accelerated capacity for molecule transport. In addition to previously known potential genetic alterations, such as elongation of telomere length and deregulation of cell cycle regulatory factors including p53, E2F1, the CDKs, and cyclins, which could possibly allow DF-1 cells to become immortal, one of the new potential regulatory factors suggested in this study is the cellular SRC (c-SRC) molecule, which is a cellular counter part of viral SRC (v-SRC) oncoprotein found in Rous sarcoma virus. The c-SRC is generally known as a protooncogene involved in regulating cellular proliferation and the mutant version of c-SRC will induce transformation. Though it is not known whether the c-SRC gene in DF-1 cells is mutated or not, the increased expression of c-SRC in DF-1 CEF cells suggests a contribution to the immortalization of the cell by prolonged activation of growth signal and the anti-apoptotic activity. Indeed, we performed functional analysis using small interfering (siRNA) to target E2F1, BRCA1, and SRC, three highly up-regulated and potentially meaningful genes for the rapid growth of DF-1 cells. As shown in Additional file 4 A-D, siRNA against E2F1 (A) marginally reduced DF-1 cell growth [2.16 fold increase in the number of cells at 3 day post transfection (dpt) compared to the number of cells at 1dpt] compared to the negative control siRNA transfection (2.49 fold increase), siRNA against BRCA1 (B) and SRC (C) showed only 1.68- and 1.56 fold increases of DF-1 cell population at 3 dpt, respectively. The marginal effect of siE2F-1 may be due to a possible compensatory effect that other E2F family genes may have on cell proliferation such as shown by E2F3 in mouse fibroloblastic cells [68]. Knock-down approaches for BRCA1 and SRC showed more significant effects on growth inhibition in DF-1 cells, similar to the result of the positive control siRNA against beta-actin (D). Additional file 5 A shows the induction of CDKN2B (known as p15INK4B) by a demethylation chemical (5-aza-2'-deoxycytidine). Additional file 5 B and C reveal the growth inhibitory effects of inducing p15INK4B on rapidly proliferating DF-1 cells compared to control groups. Further cellular, molecular, biochemical characterization of specific factors to modulate cellular characteristics for DF-1 cells remains for future studies.

## Conclusions

In this study, we have demonstrated changes in genome-wide gene expression for the immortal DF-1 CEF cell line showing rapid growth potential and chromosomal rearrangement. Taken together, the DF-1 genome sequence, which will be announced in the near future, and the differentially expressed genes characterized here provide transcriptional insights into the regulatory mechanisms for the unique characteristics observed in immortal DF-1 CEF cells.

## Methods

### Cell culture

Cell culture reagents were purchased from Invitrogen Life Technologies (Carlsbad, CA). Primary chicken CEF cells were isolated from 10 day old specific-pathogen free (SPF) chicken embryos (Charles River Laboratories, North Franklin, CT). Whole embryos were dissociated into single cell populations using 0.25% trypsin/1 mM EDTA. Cells dissociated from embryos were suspended in a Dulbecco's Modified Eagle's Medium (DMEM, 0.45% glucose) plus 10% fetal bovine serum (FBS), 100 units/ml penicillin, 100 μg/ml streptomycin, and 2 mM L-glutamine in 10 cm tissue culture dishes (Sarstedt Inc., Newton, NC). Cultured cells were grown at 39°C in a 5% CO_2 _incubator until cells reached confluent monolayers (2 to 4 days) and primary CEF cells were passaged every 3-4 days and frozen stocks of cells were prepared from each passage at a density of 3 × 10^6 ^cells and stored in liquid nitrogen. Cell freezing medium was prepared by the addition of 40% FBS to growth media supplemented with 10% DMSO. The immortal DF-1 CEF cell line was grown using the same conditions as for primary CEF cells. All procedures of handling chicken embryos, cell cultures, and DNA/RNA were approved by Institutional Biosafety Committee (IBC: protocol number: 10007) of University of Arkansas.

### Total RNA extraction

Total RNA was extracted from primary (passage 4) and DF-1 (passage 285) CEF cells using TRIzol reagent (Invitrogen Life Technologies, Carlsbad, CA) following the manufacturer's instructions. Total RNA was treated with DNase I (New England BioLabs Inc., Ipswich, MA), and RNA was re-purified by TRIzol reagent. The quality of RNA was checked by agarose gel electrophoresis fractionation (data not shown).

### Probe labeling and microarray hybridization

A two color labeling microarray system was used to compare mRNA expression between primary and DF-1 CEF cells. Fluorescently labeled complementary RNA (cRNA) probes were generated by using the Two Color Microarray Quick Labeling kit (Agilent Technologies, Palo Alto, CA) following the manufacturer's instructions. RNA Spike-in controls were used to adjust possible dye effects following the manufacturer's instructions. The Spike-in controls represent two sets of ten synthesized RNA mixtures derived from the Adenovirus E1A transcriptome with different concentrations in each set [69,70]. These Spike-in sets were mixed with either primary or DF-1 CEF samples and co-hybridized to the arrays. Briefly, 2 μg of total RNA was mixed with Spike-in controls and converted to cDNA using reverse transcriptase and oligo dT primers in which T7 promoter sequences were added. T7 RNA polymerase was used for the synthesis and labeling of cRNA with either Cy3 dye for the primary CEF control or Cy5 dye for DF-1 CEF samples. The fluorescently labeled cRNA probes were purified using the Qiagen RNeasy Mini Kit (Qiagen Inc., Valencia, CA), and the concentration, fluorescent intensities, and quality of labeled cRNA probes were determined using a Nano-drop spectrophotometer (Thermo Scientific, Wilmington, DE). An equal amount (825 ng) of Cy3 and Cy5 labeled cRNA probes were hybridized on a 4 × 44K Agilent chicken oligo microarray (array ID: 015068). The hybridized slide was washed using a commercial kit package (Agilent Technologies, Palo Alto, CA) and then scanned using a Genepix 4000B scanner (Molecular Devices Corporation, Sunnyvale, CA) with the tolerance of saturation setting of 0.005%. Four biological replicates for each cell line were conducted.

### Microarray data collection and analysis

Background-corrected red and green intensities were normalized by the local polynomial regression (loess) method. The average values of the resulting normalized expression in replicate hybridization sets were considered in the subsequent analysis. In order to identify differentially expressed genes, moderated t-statistic and its corresponding p-value based on empirical Bayes methods [71] for each gene were computed. The genes with both a p-value below 0.05 and fold change over ± 2 fold were considered as statistically different between two groups and identified as differentially expressed genes. Results were deposited into Gene Expression Omnibus (GEO; accession number: GSE29257). All analyses were implanted in Microsoft Excel and JMP Genomics (SAS Institute, Cary, NC), which is licensed to Cell and Molecular Biology Program of University of Arkansas.

### Quantitative reverse transcription-polymerase chain reaction (qPCR)

Reverse transcription was performed with 3 μg of total RNA using Superscript II reverse transcriptase and oligo dT_12-18 _primers (Invitrogen Life Technologies, Carlsbad, CA) following the manufacturer's instructions. The reverse-transcribed cDNA was diluted by 1:10 ratio and a portion (1 μl) was subjected to qPCR under the following conditions: 40 cycles of 95°C for 30 s, gene-specific annealing temperature (58 - 65°C) for 1 min, extension for 30 s at 72°C, and a final extension at 72°C for 10 min. A non-template control and endogenous loading control (chicken GAPDH) were used for the relative quantification. The differential expression in DF-1 CEF cells were calculated by the -ΔΔCT method, which is comparable to log_2 _value of differentially expressed genes, against the primary CEF counterpart [72]. Primers for qPCR were designed using Primer3 software http://frodo.wi.mit.edu/cgi-bin/primer3/primer3.cgi and were synthesized by Integrated DNA Technologies (Coralville, IA). Primer information is listed in Table 5. All qPCR reactions were performed three times.

**Table 5:** Primers used for qRT-PCR

Accession #	Forward Primer (5' → 3') Reverse Primer (5' → 3')	Gene Symbol
AB109635	GGCACCAACTTGCTACCACA GCTGCAAGAGCTGCCATTAG	HMGCR

AB196971	AGTGATGGGCGGAACAGAGT GTCTTGCTGCACACCGACTT	APCDD1

AF505881	CCAGCTACATCTCCCACCTG TCTGTTTGGGCTGGGAGTTC	SCX

AJ131110	GTGGATAGCTTGGGGACCAG AAGACTGGGAGCTGGGACTG	TWIST2

BU279212	GGCAACCATTTTGATCTTGCT CCCCTGCAAAAGCTGAAATC	CBLN2

BX929635	TCTCGCTCCTGGCAATGATA CCAGCAGCACCATCTTTGAG	NDP

BX931599	CTGTTTCCTGACCGCAGTTC AGCACAAACTCCGCCATTTT	VIPR2

BX932694	GGCCCCTTACTGGTGGTCTT AGACGGGCTTTGGATAGCAA	MPZL2

BX933478	CCTGTGCAAGGTGTCCAGTG CCCAATGGCCATACAGTTCA	MXRA5

BX933888	CTGGGATCCCTCCAGAGCTA CCATTCACTGGAGCACCAAA	C1QTNF3

BX935456	CGAGGCCATCAACTTCCTTC TCCACATGACGCACATACCC	EGLN3

BX936211	CCAGCTGTCCTCCTTGGAAT AGGGAGAGGAAGACGTGCTG	TMEM116

CK611983	ATGATGACTGGGGGCTTGTC GCAACCACTTGAGTCCGGTA	CSTA

CR385566	CGCGCTCTACGACTACATGC CTGGGTGGTGATCTCGGTCT	CLEC3B

D87992	TGCAGCACTGAGACCTGGAT CAGTTGCTGCGGATGAAGTC	ANPEP

M60853	TTTTGGCTACCAGTCCAGCA TTCGCAAGTGTTCCCCAGTA	THBS2

M64990	TGCTCCCCTGAGTACTGGAA GCCTCTGTGGGTTCAGGATT	PTGS2

M80584	TCCCACTGAGCAGCTTCTGTA CCAGAGAGATATCCGCAGCA	LUM

M87294	GGTGCTGACTTTCGCCTTGT GCCTGGTGATGAGGTTGATG	NPY

X87609	CCACCTGAGAAAAGCGACCT ACATCGACCTCTGCCAACCT	FST

NM_204305	GGCACTGTCAAGGCTGAGAA TGCATCTGCCCATTTGATGT	chGAPDH

The first column indicates the NCBI accession number for designated genes, and the second column shows sequences for the forward and reverse primers. The gene symbols are provided in the third column.

### Bioinformatics

Functional interpretation of differentially expressed genes was analyzed in the context of gene ontology and molecular networks using the Ingenuity Pathways Analysis (IPA; Ingenuity Systems^®; ^http://www.ingenuity.com). Since IPA is based on human and mouse bioinformatics, functionalities for differentially expressed genes in the chicken were interpreted based primarily on mammalian biological mechanisms. The differentially expressed genes were compared to genetic categories in the IPA database, and ranked according to p-values [73]. Since the size of the created gene network could potentially be enormous, the number of molecules in the network was set to the limit of 35, leaving only the most important ones based on the number of connections for each focus gene (focus genes = a subset of uploaded significant genes having direct interactions with other genes in the database) to other significant genes [74].

## Authors' contributions

BWK and JYL designed the experiments, performed the experiments, analyzed the data, and wrote the manuscript. WB and KL contributed to the interpretation of the bioinformatics analysis and manuscript editing. JL analyzed the qPCR assay and DNF prepared DF-1 cells and edited the manuscript preparation. All authors read and approved the final manuscript.

## Supplementary Material

Additional file 1 **List of entire 3876 DE genes before sorting by IPA**. The values indicate Log_2 _fold changes. The Agilent ID, gene symbol, gene name, GenBank accession numbers, chromosomal region, cytoband, GO ID, and oligo sequence on the array were provided. Click here for file

Additional file 2 **List of 902 DE genes identified by IPA database**. The values indicate Log_2 _fold changes. The gene symbol, gene name, GenBank accession numbers, cellular locations, and molecule types were provided. Click here for file

Additional file 3 **List of focus molecules in gene networks**. Gene symbols and GenBank accession numbers were displayed for the illustrations of network analysis. Only focus molecules, which were elected as differentially expressed genes from microarray analysis, include GenBank accession numbers, while accession numbers for reference molecules were not shown in the table. Click here for file

Additional file 4 **DF-1 cell growth responding to siRNA for E2F-1, BRCA1, and SRC**. Each of four small siRNAs to target chE2F1 (A), chBRCA1 (B), chSRC (C), and chBeta-actin (D), in addition to a negative control siRNA were synthesized by Integrated DNA Technology Inc. (Coralville, IA). One million DF-1 cells were transfected with 300 pmole of each siRNA using Lipofectamine reagent (Invitrogen Life Technologies, Carlsbad, CA). Transfected cells were collected at 1 and 3 days post transfection (dpt), total cell numbers were counted, and the growth rates were determined by ratio of cell numbers at 3dpt and cell numbers at 1dpt. Results were compared to a negative control. Results of the most effective siRNA for each target were displayed. The siRNA for chBeta-actin was used as positive control to suppress DF-1 cell growth. Click here for file

Additional file 5 **DF-1 cell growth responding to 5-aza-2'-deoxycytidine treatment for the induction of p15INK4B**. A 2 μM concentration of 5-aza-2'-deoxycytidine (5-aza), which is a demethylation chemical, was used to treat 1 million DF-1 cells, cells which were collected at 1, 2, 3, and 4 days post treatment. The mRNA expression of p15INK4B was determined by qRT-PCR at designated time points (A); cell morphology was visualized by phase-contrast microscopy (400 ×; B); and cell numbers were counted to determine growth rates (C). Click here for file
