# Supplementary material for: Genome-wide differential gene expression in immortalized DF-1 chicken embryo fibroblast cell line
Source: BMC Genomics. 2011 Nov 23;12:571. doi: 10.1186/1471-2164-12-571 (PMC3258366; doi:10.1186/1471-2164-12-571)
Supplement: Additional file 3 — List of focus molecules in gene networks. Gene symbols and GenBank accession numbers were displayed for the illustrations of network analysis. Only focus molecules, which were elected as differentially expressed genes from microarray analysis, include GenBank accession numbers, while accession numbers for reference molecules were not shown in the table. [file 1471-2164-12-571-S3.DOC]

**Network #1 - Cell Cycle, DNA Replication, Recombination, and Repair, Cellular Assembly and Organization**

| Symbol | Entrez Gene Name | GenBank | Log Ratio | p-value |
| --- | --- | --- | --- | --- |
| ABCC6 | ATP-binding cassette, sub-family C (CFTR/MRP), member 6 | CR523237 | 1.25 | 9.07E-5 |
| ARHGEF4 | Rho guanine nucleotide exchange factor (GEF) 4 | CR387262 | -1.84 | 4.52E-4 |
| ATP1B1 | ATPase, Na+/K+ transporting, beta 1 polypeptide | J02787 | 1.72 | 8.65E-7 |
| ATP5S | ATP synthase, H+ transporting, mitochondrial F0 complex, subunit s (factor B) | BX932166 | 1.23 | 3.86E-5 |
| ATPase |  |  |  |  |
| BARD1 | BRCA1 associated RING domain 1 | AJ720093 | 2.09 | 2.67E-7 |
| Basc |  |  |  |  |
| BRCA1 | breast cancer 1, early onset | AF355273 | 1.65 | 6.06E-4 |
| CCNG1 | cyclin G1 | BX935990 | -1.99 | 3.67E-8 |
| DNA polymerase |  |  |  |  |
| E2F1 | E2F transcription factor 1 | X89245 | 1.16 | 5.09E-5 |
| EVL | Enah/Vasp-like | AJ720855 | -1.10 | 1.40E-4 |
| FEN1 | flap structure-specific endonuclease 1 | AJ719700 | 2.23 | 6.53E-6 |
| Jnk |  |  |  |  |
| KATNA1 | katanin p60 (ATPase-containing) subunit A 1 | BX934536 | 1.45 | 3.85E-9 |
| MED17 | mediator complex subunit 17 | AJ720853 | 1.72 | 3.65E-6 |
| MT2A | metallothionein 2A | X06749 | -1.55 | 2.89E-4 |
| MTF1 | metal-regulatory transcription factor 1 | AJ719787 | -1.22 | 2.87E-5 |
| NBN | nibrin | AF230342 | 1.07 | 9.61E-5 |
| POLD3 | polymerase (DNA-directed), delta 3, accessory subunit | AJ720256 | 1.12 | 1.72E-6 |
| PRIM2 | primase, DNA, polypeptide 2 (58kDa) | AJ719578 | 1.77 | 1.52E-8 |
| RAD17 | RAD17 homolog (S. pombe) | AJ720361 | 1.12 | 7.01E-6 |
| RAD51 | RAD51 homolog (RecA homolog, E. coli) (S. cerevisiae) | L09655 | 1.56 | 3.42E-7 |
| RAD52 | RAD52 homolog (S. cerevisiae) | BX932349 | 1.23 | 1.99E-5 |
| RAD54B | RAD54 homolog B (S. cerevisiae) | AF178529 | 1.41 | 0.002 |
| RAD54L | RAD54-like (S. cerevisiae) | U92461 | 1.07 | 1.51E-4 |
| Rfc |  |  |  |  |
| RFC2 | replication factor C (activator 1) 2, 40kDa | U12438 | 2.24 | 1.66E-8 |
| RFC3 | replication factor C (activator 1) 3, 38kDa | AJ720394 | 2.28 | 3.30E-8 |
| RFC4 | replication factor C (activator 1) 4, 37kDa | BX931624 | 1.15 | 3.28E-5 |
| RPA |  |  |  |  |
| SLC19A1 | solute carrier family 19 (folate transporter), member 1 | BX934894 | 1.18 | 1.62E-4 |
| STAT1/3/5 dimer |  |  |  |  |
| TFAP2B | transcription factor AP-2 beta (activating enhancer binding protein 2 beta) | AF065140 | 2.42 | 1.27E-7 |
| TIPIN | TIMELESS interacting protein | AJ851484 | 1.69 | 3.41E-7 |

**Network #2- DNA Replication, Recombination, and Repair, Cell Cycle, Cellular Assembly and Organization**

| Symbol | Entrez Gene Name | GenBank | Log Ratio | p-value |
| --- | --- | --- | --- | --- |
| 20s proteasome |  |  |  |  |
| AATF | apoptosis antagonizing transcription factor | AJ720758 | 1.42 | 0.00058 |
| APC |  |  |  |  |
| CASP3 | caspase 3, apoptosis-related cysteine peptidase | AF083029 | -1.272 | 0.00146 |
| CASP3/6/7 |  |  |  |  |
| CEP55 | centrosomal protein 55kDa | CR385518 | 1.36 | 9.5E-05 |
| COL11A1 | collagen, type XI, alpha 1 | M88593 | -5.332 | 9.1E-08 |
| E2f |  |  |  |  |
| GMNN | geminin, DNA replication inhibitor | AJ720138 | 2.03 | 5.4E-06 |
| KIF23 | kinesin family member 23 | AJ720929 | 1.17 | 1.2E-06 |
| MCM2 | minichromosome maintenance complex component 2 | CR338832 | 1.17 | 6.2E-05 |
| MCM3 | minichromosome maintenance complex component 3 | AJ719352 | 1.14 | 3.4E-08 |
| MCM5 | minichromosome maintenance complex component 5 | AJ720074 | 1.29 | 7.1E-07 |
| MCM6 | minichromosome maintenance complex component 6 | AJ720172 | 1.67 | 1.8E-06 |
| NASP | nuclear autoantigenic sperm protein (histone-binding) | AJ719339 | 1.14 | 0.0006 |
| ORC1L | origin recognition complex, subunit 1-like (yeast) | AJ719459 | 2.11 | 3.1E-06 |
| ORC2L | origin recognition complex, subunit 2-like (yeast) | AJ720422 | 1.84 | 6.4E-06 |
| ORC6L | origin recognition complex, subunit 6 like (yeast) | BX932635 | 1.47 | 1.6E-07 |
| PARP |  |  |  |  |
| PARP4 | poly (ADP-ribose) polymerase family, member 4 | BX932696 | 1.19 | 4.4E-06 |
| PHB (includes EG:5245) | prohibitin | CR387706 | 1.18 | 0.00047 |
| PLK1 | polo-like kinase 1 (Drosophila) | AJ720598 | 1.08 | 6.7E-05 |
| PRC1 | protein regulator of cytokinesis 1 | AJ851410 | 1.50 | 1.1E-05 |
| PSME3 | proteasome (prosome, macropain) activator subunit 3 (PA28 gamma; Ki) | AJ719711 | 1.12 | 1.2E-06 |
| Rb |  |  |  |  |
| SFRP1 | secreted frizzled-related protein 1 | AJ404652 | -1.27 | 0.00153 |
| SMARCA2 | SWI/SNF related, matrix associated, actin dependent regulator of chromatin, subfamily a, member 2 | X91638 | -1.15 | 0.00036 |
| SNCG | synuclein, gamma (breast cancer-specific protein 1) | AF253513 | 1.03 | 0.00085 |
| SWI-SNF |  |  |  |  |
| Thymidine Kinase |  |  |  |  |
| TK1 | thymidine kinase 1, soluble | X04353 | 1.23 | 7.9E-07 |
| TMEM126A | transmembrane protein 126A | BX931781 | 1.43 | 6.8E-06 |
| TPX2 | TPX2, microtubule-associated, homolog (Xenopus laevis) | AB101006 | 1.71 | 1E-06 |
| TRIB2 | tribbles homolog 2 (Drosophila) | AY247742 | -1.13 | 5.1E-07 |
| TYMS | thymidylate synthetase | BX932834 | 2.05 | 2.4E-08 |

**Network #3- Cancer, Cardiovascular System Development and Function, Organismal Development**

| Symbol | Entrez Gene Name | GenBank | Log Ratio | p-value |
| --- | --- | --- | --- | --- |
| Ap1 |  |  |  |  |
| CaMK-II/IV |  |  |  |  |
| CAMK4 | calcium/calmodulin-dependent protein kinase IV | BX950684 | -1.41 | 2.61E-05 |
| Casein |  |  |  |  |
| CASP2 | caspase 2, apoptosis-related cysteine peptidase | U64963 | 1.08 | 1.67E-04 |
| CASP8 | caspase 8, apoptosis-related cysteine peptidase | AY057939 | 1.09 | 5.88E-05 |
| CLSTN1 | calsyntenin 1 | AJ289017 | -1.59 | 6.68E-06 |
| COL4A1 | collagen, type IV, alpha 1 | AF239838 | -5.93 | 3.38E-08 |
| CST3 | cystatin C | J05077 | -1.31 | 2.83E-04 |
| CTSS | cathepsin S | AJ719318 | -1.69 | 1.43E-08 |
| DLX5 | distal-less homeobox 5 | U25274 | -1.49 | 1.75E-04 |
| DNER | delta/notch-like EGF repeat containing | BX935146 | -2.91 | 2.48E-07 |
| DTL | denticleless homolog (Drosophila) | AJ720316 | 1.25 | 1.12E-07 |
| EMP1 | epithelial membrane protein 1 | BX930381 | -1.01 | 3.76E-06 |
| Glutathione peroxidase |  |  |  |  |
| glutathione transferase |  |  |  |  |
| GPX7 | glutathione peroxidase 7 | BX933973 | -1.68 | 1.89E-05 |
| GPX8 | glutathione peroxidase 8 (putative) | BX932298 | -1.77 | 1.07E-06 |
| GST |  |  |  |  |
| GSTA4 | glutathione S-transferase alpha 4 | AF133251 | -2.23 | 7.27E-07 |
| GSTO1 | glutathione S-transferase omega 1 | BX934375 | 2.61 | 9.19E-05 |
| GSTT1 | glutathione S-transferase theta 1 | U13676 | -2.76 | 4.46E-05 |
| GSTZ1 (includes EG:2954) | glutathione transferase zeta 1 | BX935175 | -1.56 | 3.52E-07 |
| hCG |  |  |  |  |
| IMMP2L | IMP2 inner mitochondrial membrane peptidase-like (S. cerevisiae) | BX930706 | -2.18 | 2.20E-06 |
| LAP3 | leucine aminopeptidase 3 | AJ720335 | -1.14 | 6.13E-05 |
| LSAMP | limbic system-associated membrane protein | Z94720 | -2.89 | 2.35E-08 |
| NTM | neurotrimin | AF292935 | -1.72 | 1.10E-03 |
| PBK | PDZ binding kinase | BX930018 | 1.11 | 1.76 E-04 |
| PEPD | peptidase D | AJ720071 | 1.06 | 7.81E-06 |
| peptidase |  |  |  |  |
| PKIA | protein kinase (cAMP-dependent, catalytic) inhibitor alpha | U19496 | -3.34 | 5.73E-11 |
| PRNP | prion protein | M61145 | -4.91 | 3.24E-08 |
| Smad2/3 |  |  |  |  |
| SMARCA1 | SWI/SNF related, matrix associated, actin dependent regulator of chromatin, subfamily a, member 1 | CR406600 | 1.12 | 1.19E-03 |

**Network #4- Molecular Transport, Tissue Morphology, Cell Cycle**

| Symbol | Entrez Gene Name | GenBank | Log Ratio | p-value |
| --- | --- | --- | --- | --- |
| Adaptor protein 1 |  |  |  |  |
| AP1S3 (includes EG:130340) | adaptor-related protein complex 1, sigma 3 subunit | BX931554 | -3.62 | 4.61E-08 |
| AQP1 | aquaporin 1 (Colton blood group) | BX933585 | 2.57 | 5.09E-05 |
| EGLN3 | egl nine homolog 3 (C. elegans) | BX935456 | -3.04 | 7.45E-07 |
| EGR1 | early growth response 1 | AJ719635 | -1.25 | 2.89E-05 |
| EZR | ezrin | AB019790 | 1.11 | 1.16E-04 |
| FGFBP1 | fibroblast growth factor binding protein 1 | BX930139 | -2.80 | 8.20E-07 |
| FOXM1 | forkhead box M1 | BX931138 | 1.71 | 1.18E-06 |
| GOT |  |  |  |  |
| GTF3A | general transcription factor IIIA | CR352757 | 1.09 | 1.54E-05 |
| HELLS | helicase, lymphoid-specific | AJ851608 | 1.33 | 1.89E-05 |
| HES1 | hairy and enhancer of split 1, (Drosophila) | AY225440 | -1.09 | 2.32E-05 |
| Ige |  |  |  |  |
| IgG |  |  |  |  |
| IL16 | interleukin 16 (lymphocyte chemoattractant factor) | AJ721031 | -3.63 | 5.66E-08 |
| Immunoglobulin |  |  |  |  |
| KIF11 | kinesin family member 11 | AJ719314 | 1.21 | 2.97E-04 |
| Mek |  |  |  |  |
| MELK | maternal embryonic leucine zipper kinase | AJ719849 | 1.10 | 2.56E-05 |
| MEOX2 | mesenchyme homeobox 2 | AJ401088 | -4.78 | 4.21E-10 |
| NDC80 | NDC80 homolog, kinetochore complex component (S. cerevisiae) | AJ719646 | 1.29 | 1.15E-04 |
| NFIL3 | nuclear factor, interleukin 3 regulated | AF335427 | -1.56 | 9.56E-04 |
| NRG1 | neuregulin 1 | L11264 | 2.16 | 1.01E-05 |
| NUF2 | NUF2, NDC80 kinetochore complex component, homolog (S. cerevisiae) | AJ720907 | -1.99 | 6.41E-09 |
| PLS3 | plastin 3 | AJ720945 | -1.04 | 2.72E-05 |
| PTGS2 | prostaglandin-endoperoxide synthase 2 (prostaglandin G/H synthase and cyclooxygenase) | M64990 | -6.78 | 1.11E-10 |
| SERPINE2 | serpin peptidase inhibitor, clade E (nexin, plasminogen activator inhibitor type 1), member 2 | BX934893 | -4.23 | 6.37E-09 |
| SMOX | spermine oxidase | BX932163 | -1.19 | 0.002318 |
| SPC25 | SPC25, NDC80 kinetochore complex component, homolog (S. cerevisiae) | BX932394 | 1.00 | 1.91E-04 |
| SRC |  |  |  |  |
| TBC1D8 | TBC1 domain family, member 8 (with GRAM domain) | BX931824 | 1.36 | 4.47E-07 |
| TP53INP1 | tumor protein p53 inducible nuclear protein 1 | AJ851440 | -1.45 | 8.20E-08 |
| Vegf |  |  |  |  |
| VIP | vasoactive intestinal peptide | U09350 | 2.44 | 0.008539 |
| VIPR2 | vasoactive intestinal peptide receptor 2 | BX931599 | -2.90 | 5.36E-06 |

**Network #5- Cellular Assembly and Organization, Developmental Disorder, Skeletal and Muscular Disorders**

| Symbol | | Entrez Gene Name | GenBank | Log Ratio | p-value |
| --- | --- | --- | --- | --- | --- |
| Actin | |  |  |  |  |
| AFAP1 | | actin filament associated protein 1 | L20302 | -1.12 | 3.50E-05 |
| AGRN | | agrin | M94271 | -3.35 | 1.17E-07 |
| Alpha Actinin | |  |  |  |  |
| Alpha catenin | |  |  |  |  |
| Cadherin | |  |  |  |  |
| Calcineurin A | |  |  |  |  |
| CAV2 | | caveolin 2 | BX934522 | 1.31 | 3.27E-05 |
| Caveolin | |  |  |  |  |
| CDH11 | | cadherin 11, type 2, OB-cadherin (osteoblast) | AF055342 | -4.27 | 4.29E-09 |
| Clathrin | |  |  |  |  |
| CLDN5 | | claudin 5 | CR354218 | -3.14 | 7.88E-06 |
| CSRP1 | | cysteine and glycine-rich protein 1 | CR354109 | -1.30 | 5.13E-05 |
| CSRP2 | | cysteine and glycine-rich protein 2 | X84264 | -2.32 | 1.65E-05 |
| CTNNA2 | | catenin (cadherin-associated protein), alpha 2 | D11090 | -3.41 | 7.79E-07 |
| DTX2 | | deltex homolog 2 (Drosophila) | CR387657 | 1.33 | 1.24E-06 |
| dystroglycan |  | |  |  |  |
| KIF3B | | kinesin family member 3B | CR405855 | 1.46 | 1.27E-07 |
| MALL | | mal, T-cell differentiation protein-like | BX929438 | -3.67 | 1.38E-08 |
| MAP2 | | microtubule-associated protein 2 | AY524980 | -2.02 | 4.54E-06 |
| MARCKSL1 | | MARCKS-like 1 | AJ720014 | -2.21 | 9.02E-07 |
| MTMR2 | | myotubularin related protein 2 | AJ720683 | 1.50 | 6.43E-06 |
| NCALD | | neurocalcin delta | U91630 | 1.61 | 6.58E-05 |
| NEO1 | | neogenin homolog 1 (chicken) | U07644 | 1.09 | 5.64E-05 |
| PP1-C | |  |  |  |  |
| Rab5 | |  |  |  |  |
| RALGAPB | | Ral GTPase activating protein, beta subunit (non-catalytic) | AJ851653 | 1.06 | 1.14E-05 |
| ROR1 | | receptor tyrosine kinase-like orphan receptor 1 | AJ620298 | -1.25 | 3.36E-04 |
| SEPN1 (includes EG:57190) | | selenoprotein N, 1 | CR352801 | -2.02 | 6.85E-08 |
| SGCB | | sarcoglycan, beta (43kDa dystrophin-associated glycoprotein) | AJ720367 | -1.13 | 4.19E-06 |
| SGCD | | sarcoglycan, delta (35kDa dystrophin-associated glycoprotein) | CR405962 | -1.16 | 5.02E-06 |
| SRC | | v-src sarcoma (Schmidt-Ruppin A-2) viral oncogene homolog (avian) | S43579 | 1.52 | 5.10E-06 |
| TJP2 | | tight junction protein 2 (zona occludens 2) | AF085184 | -1.72 | 7.91E-06 |
| TRPC1 | | transient receptor potential cation channel, subfamily C, member 1 | AY657006 | -1.07 | 3.37E-06 |
| ZP2 | | zona pellucida glycoprotein 2 (sperm receptor) | AB197938 | 1.19 | 0.003103 |
