# Supplementary material for: Genome-wide differential gene expression in immortalized DF-1 chicken embryo fibroblast cell line
Source: BMC Genomics. 2011 Nov 23;12:571. doi: 10.1186/1471-2164-12-571 (PMC3258366; doi:10.1186/1471-2164-12-571)
Supplement: Additional file 4 — DF-1 cell growth responding to siRNA for E2F-1, BRCA1, and SRC. Each of four small siRNAs to target chE2F1 (A), chBRCA1 (B), chSRC (C), and chBeta-actin (D), in addition to a negative control siRNA were synthesized by Integrated DNA Technology Inc. (Coralville, IA). One million DF-1 cells were transfected with 300 pmole of each siRNA using Lipofectamine reagent (Invitrogen Life Technologies, Carlsbad, CA). Transfected cells were collected at 1 and 3 days post transfection (dpt), total cell numbers were counted, and the growth rates were determined by ratio of cell numbers at 3dpt and cell numbers at 1dpt. Results were compared to a negative control. Results of the most effective siRNA for each target were displayed. The siRNA for chBeta-actin was used as positive control to suppress DF-1 cell growth. [file 1471-2164-12-571-S4.PPT]

## Slide 1
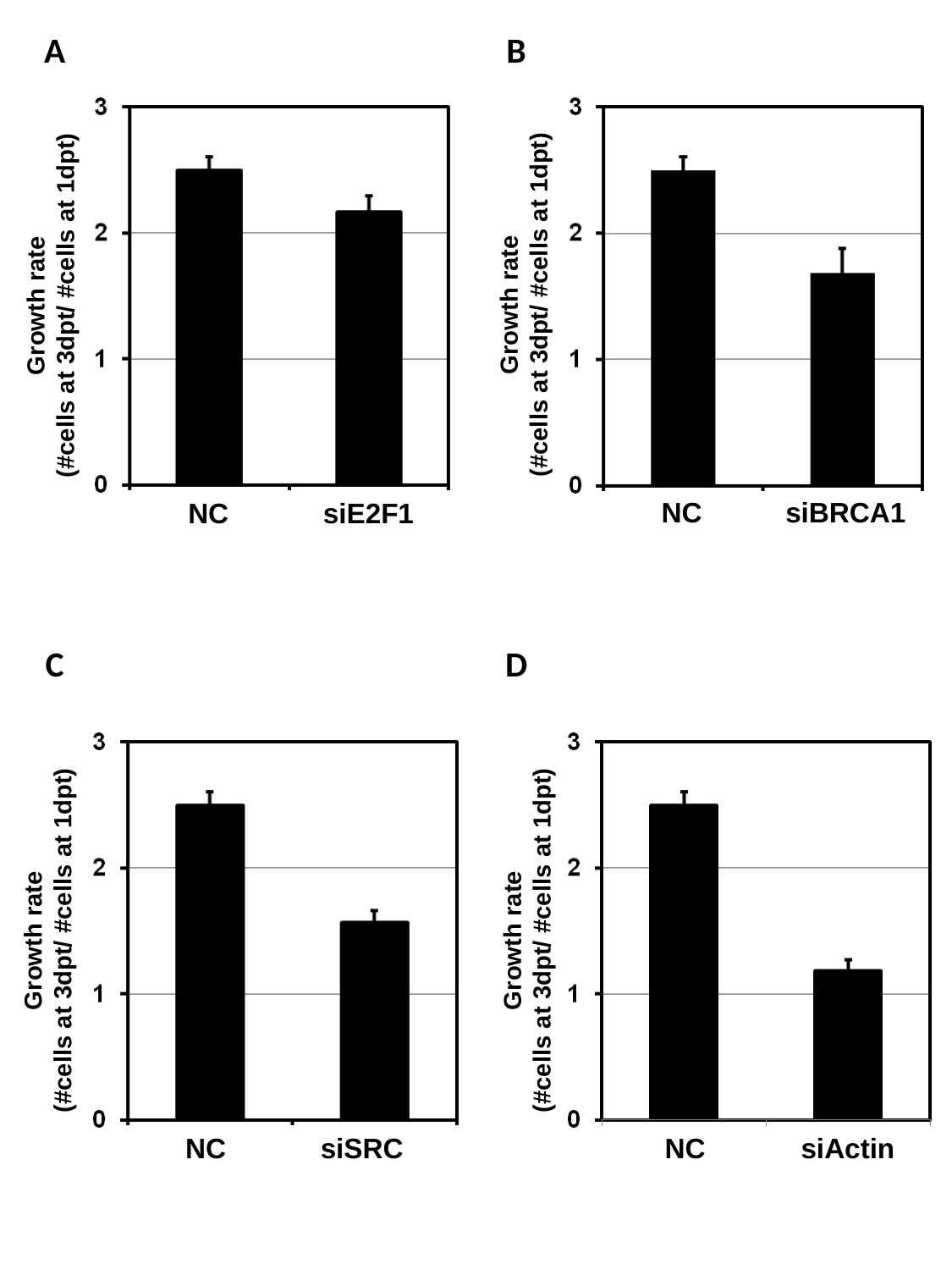

A
B
 Growth rate
(#cells at 3dpt/ #cells at 1dpt)
 Growth rate
(#cells at 3dpt/ #cells at 1dpt)
NC
siBRCA1
NC
siE2F1
C
D
 Growth rate
(#cells at 3dpt/ #cells at 1dpt)
 Growth rate
(#cells at 3dpt/ #cells at 1dpt)
NC
siSRC
NC
siActin
