# Supplementary material for: Genome-wide differential gene expression in immortalized DF-1 chicken embryo fibroblast cell line
Source: BMC Genomics. 2011 Nov 23;12:571. doi: 10.1186/1471-2164-12-571 (PMC3258366; doi:10.1186/1471-2164-12-571)
Supplement: Additional file 5 — DF-1 cell growth responding to 5-aza-2'-deoxycytidine treatment for the induction of p15INK4B. A 2 μM concentration of 5-aza-2'-deoxycytidine (5-aza), which is a demethylation chemical, was used to treat 1 million DF-1 cells, cells which were collected at 1, 2, 3, and 4 days post treatment. The mRNA expression of p15INK4B was determined by qRT-PCR at designated time points (A); cell morphology was visualized by phase-contrast microscopy (400 ×; B); and cell numbers were counted to determine growth rates (C). [file 1471-2164-12-571-S5.PPT]

## Slide 1
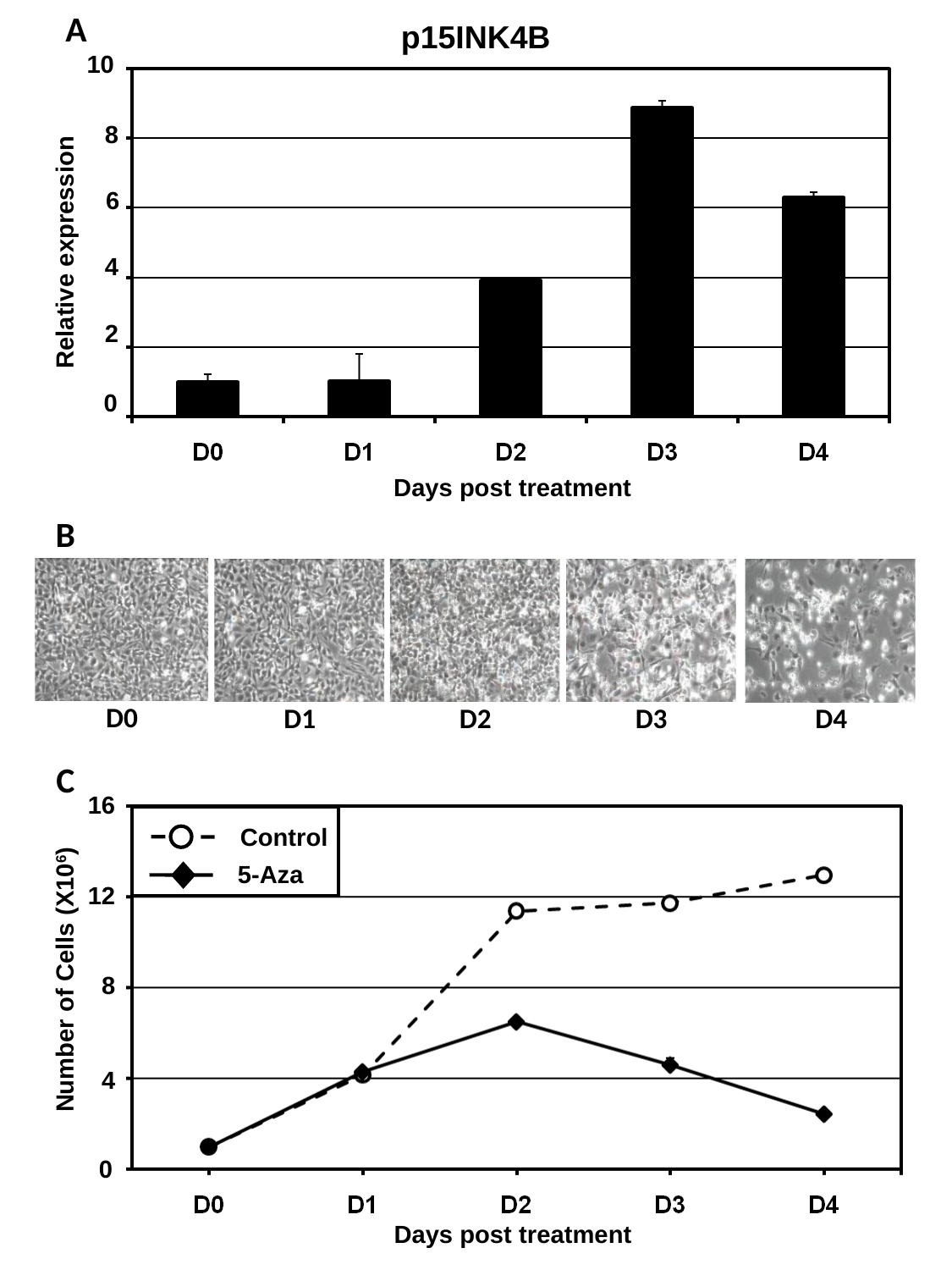

A
p15INK4B
 10
 8
6
Relative expression
 4
 2
 0
Days post treatment
B
D0
D4
D1
D2
D3
C
 16
Control
5-Aza
12
 Number of Cells (X106)
 8
 4
 0
Days post treatment
